# An Extensive Review on Preclinical and Clinical Trials of Oncolytic Viruses Therapy for Pancreatic Cancer

**DOI:** 10.3389/fonc.2022.875188

**Published:** 2022-05-24

**Authors:** Maryum Nisar, Rehan Zafar Paracha, Sidra Adil, Sumair Naseem Qureshi, Hussnain Ahmed Janjua

**Affiliations:** ^1^ School of Interdisciplinary Engineering & Sciences (SINES), National University of Sciences and Technology (NUST), Islamabad, Pakistan; ^2^ Shifa College of Medicine, Islamabad, Pakistan; ^3^ Atta-ur-Rahman School of Applied Biosciences (ASAB), National University of Sciences and Technology (NUST), Sector H-12, Islamabad, Pakistan

**Keywords:** pancreatic cancer, immunotherapy, oncolytic virus therapy, Adenovirus, recombinant virus, clinical trials, therapy-resistance, preclinical studies

## Abstract

Chemotherapy resistance and peculiar tumor microenvironment, which diminish or mitigate the effects of therapies, make pancreatic cancer one of the deadliest malignancies to manage and treat. Advanced immunotherapies are under consideration intending to ameliorate the overall patient survival rate in pancreatic cancer. Oncolytic viruses therapy is a new type of immunotherapy in which a virus after infecting and lysis the cancer cell induces/activates patients’ immune response by releasing tumor antigen in the blood. The current review covers the pathways and molecular ablation that take place in pancreatic cancer cells. It also unfolds the extensive preclinical and clinical trial studies of oncolytic viruses performed and/or undergoing to design an efficacious therapy against pancreatic cancer.

## 1 Introduction

Pancreatic cancer is one of the wearisome malignancies to manage and treat, with the world’s seventh-highest mortality rate (1, 2). A total of 495,773 new cases along with 466,003 deaths due to pancreatic cancer were recorded in the year 2020 (1). In the U.S, it is the 3rd prime reason for deaths caused by cancer (1). It is expected to surpass breast cancer and become the 2nd most deadly type of cancer by 2030 (3). It develops in the pancreas and is divided into two types based on the tissues in which cancer develops: pancreatic ductal adenocarcinoma (PDAC) and pancreatic neuroendocrine tumor. The PDAC which develops in the pancreatic duct or exocrine part of the pancreas accounts for 95% of pancreatic cancer cases, hence the most prevalent and fatal type. While the pancreatic neuroendocrine tumor grows in the endocrine region of the pancreas and accounts for 5% of pancreatic cancer cases (2, 4).

The fatal nature of pancreatic cancer strongly depends on its high tendency to metastasize and late diagnosis (4). Surgery is the only potential therapeutic option for pancreatic cancer. Only a small number of patients (less than 20%) are authorized for surgery at diagnosis and only 20% to 30% of those survive for 5 years (3, 5). Approximately 80% of patients diagnosed at a later stage of pancreatic cancer do not have effective treatment options available (5). The unresectable locally advanced pancreatic cancer (LAPC) patients have a survival rate of <5%. By the time of diagnosis, approximately one-third of patients exhibit metastasis, thus bringing about a low survival rate of 2.9% (6, 7). Abdominal pain, nausea, mid-back pain, weight loss, obstructive jaundice (8), postprandial, and/or gastrointestinal (GI) bleeding are the disease related symptoms that pancreatic cancer patients develop in later stages. Venous thromboembolism (VTE) is also very prevalent in pancreatic cancer patients (6).

Despite many efforts to develop an effective treatment to increase the overall survival rate and reduce patient de-conditioning, only 9% of patients survive within the first 5 years of diagnosis. To date combination regimen is recommended for LAPC patients, combining chemotherapy, radiation therapy, and nano-particle. The types of combinational regimens given to LAPC patients are chemoradiotherapy (CRT) or stereotactic body radiation therapy (SBRT), but there is no strong suggestion regarding which of these regimens should be used over the other (6). Radiation therapy (CRT) is recommended to the patient if he shows stable or progression-free disease after the first 6 months of chemotherapy (6, 9). For LAPC patients Fluoropyrimidines or Gemcitabine are simultaneously administered along with the radiotherapy (9). Only 40% to 60% of patients given CRT showed 1-year progression control survival because of the radioresistant nature of pancreatic cancer (10). The toxicity profile of all combinational regimens was analyzed initially, a high incidence rate of patient de-conditioning is reported with acute GI toxicity, and commonly GI ulceration. LAPC patients with very low to no benefit from first-line treatment are given the medication regarding Metastatic Pancreatic Cancer Treatment Guideline (6). Above 30% to 50% of LAPC patients develop a metastatic state within 3 months of diagnosis even with the treatment (6). Patients diagnosed with metastatic PDAC have a median life expectancy of <1 year. FOLFIRINOX (folinic acid, 5-fluorouracil, irinotecan, and oxaliplatin) is the only most effective chemotherapy so far for such patients, which only enhances the survival span to 11.1 months (11, 12).

In the current review, firstly, we provided a brief introduction to pancreatic cancer, genetic changes & impaired pathways leading to cancer development, and a general overview of immune therapies. Further, an extensive review and discussion on oncolytic viruses therapy are provided. We have also explored the possibility of using natural compounds for the treatment of cancer. For the current review, Pubmed is explored on February 10, 2022, using the keywords Oncolytic viruses plus pancreatic cancer, and searched for all papers published from the year 2000 to date. The current review covers all clinical trial studies that have been published between this time frame (from the year 2000 to date), while the studies covering preclinical trials are selected that have been published over the last five years (from the year 2016 to 2022).

## 2 Molecular Nature of Pancreatic Cancer That Results in Therapies Failure

Various genetic alterations resulting in pancreatic cancer make treatment challenging even with targeted therapies (13). Numerous studies have reported that pancreatic cancer is vastly enriched with the cancer stem cells (CSCs) population. CSCs known as a subpopulation of tumor cells might contribute to tumor metastasis and relapses (14). This high CSC enrichment leads to chemotherapies resistance; hence, this resistant nature resulted in disease recur (13). Epithelial to mesenchymal transition (EMT) is also a significant factor to be considered in pancreatic cancer (13). In EMT, the epithelial cells undergo both the genotypic and phenotypic transitions to attain the mesenchymal phenotype. In contrast to the epithelial phenotype, the mesenchymal phenotype is known for its properties like apoptosis resistance, the ability to migrate and invade (15, 16). EMT is associated with metastasis, tumor progression, and production of CSC which eventually results in treatment resistance in various cancer types (13, 17, 18), including pancreatic cancer (13). EMT is also linked with a poor prognosis of pancreatic cancer (16). Therefore, cancer cell EMT is a crucial factor to consider in pancreatic cancer therapy design (13).

### 2.1 Mutational Landscape and Disrupted Pathways in Pancreatic Cancer

Whole genome sequencing analysis was performed in various studies to comprehend the mutational landscape of pancreatic cancer. A genetic analysis reported an average of 63 mutations per pancreatic cancer patient (19). Another extensive genetic analysis discovered an average of 119 somatic variations per pancreatic cancer patient (20). Interestingly these mutations are associated with 12 signaling pathways that are disrupted in 67-100% of the tumors (20). The triggering of certain signaling pathways [e.g., P13K/Akt (21), MAPK and TGF-β (22), hypoxia, WNT, Notch (16)], the expressions of miRNAs [e.g., miR-10b, miR-210, miR-577, miR-1207-5p, miR-5188 (23), miR‐103/107, miR‐9, miR‐181a (24)], and EMT transcription factors [e.g., Prrx1, Snail1/2, Twist1, ZEB1/2 (16, 24)] initiate the cancer cell modification from epithelial to mesenchymal phenotype. The major genetic mutations reported in pancreatic cancer patients include K-Ras, CDKN2A, TP53, SMAD4, and BRCA (BRCA1/BRCA2) genes (25, 26). 

#### 2.1.1 Dysregulation of miRNAs

MicroRNAs (miRNAs) are small non-coding RNAs (approx 20-25 nt in length), that regulate the translation of target mRNA. The regulatory role of miRNAs is crucial for the regular/normal signaling in a healthy cell (27). The disruption in miRNAs causes various diseases including pancreatic cancer. To date numerous differentially expressed miRNAs are reported to play a pivotal role in pancreatic cancer progression related pathways or processes like metastasis, drug resistance, cancer stemness (23, 28). Over expressed miR-301 regulates the EMT and causes gemcitabine resistance by suppressing the expression of E-cadherin in pancreatic cancer cells (29). Likewise, aberrantly expressed miR-296-5p targets and downregulates BOK (apoptosis regulating gene), facilitates EMT, cancer invasion, and drug resistance (23). Numerous other overexpressed miRNAs which facilitate the EMT associated signaling include miR-103/107, miR-9, miR-181a (28). The Notch signaling has a crucial role in pancreatic cancer progression by promoting EMT associated signaling. The overexpressed miR-21 enhances while Let-7 and miR-200 family miRNAs inhibit Notch signaling. The downregulation of Let-7 and miR-200 is identified in pancreatic cancer cells (30).

Multiple miRNAs with tumor suppressive function are reported to be downregulated in pancreatic cancer. List of miRNAs includes miR-148a, miR-200 family, miR-509-5p or miR-1243, Let-7, 203, miR-125a-3p, miR-31, miR-210 e.t.c. The miR-200b, miR-509-5p or miR-1243 expression enhance chemosensitivity by targeting and suppressing EMT related genes (23, 29, 31). The miR-200 and miR-203 reduce the chemoresistance in the pancreatic cancer cell, but overexpressed ZEB1 suppresses these miRNAs. (32) elucidate that the drug resistance effect of ZEB1 can be hindered by class I HDAC inhibitor mocetinostat, and can induce sensitivity against chemotherapy. Similarly overexpressed miR-125a-3p suppresses EMT by inhibiting Fyn gene expression, but in pancreatic cancer cell downregulation of miR-125a-3p is reported. (33) elucidate that cancer cells with overexpressed miR-125a-3p exhibit increased chemosensitivity. The miR-148a targets CCKBR and Bcl-2, decrease cell proliferation and perform a pro-apoptosis function. The underexpression of miR-148a is identified in pancreatic cancer cells (34). The miR-148a along with other miRNAs like miR-141, miR-200 family, miR-216a, miR-217, and miR-375 are enriched in the pancreas but their expression level decreases in pancreatic cancer (35).

### 2.2 Signaling Pathways That Regulate Pancreatic Cancer EMT & CSCs

**Figure 1:**
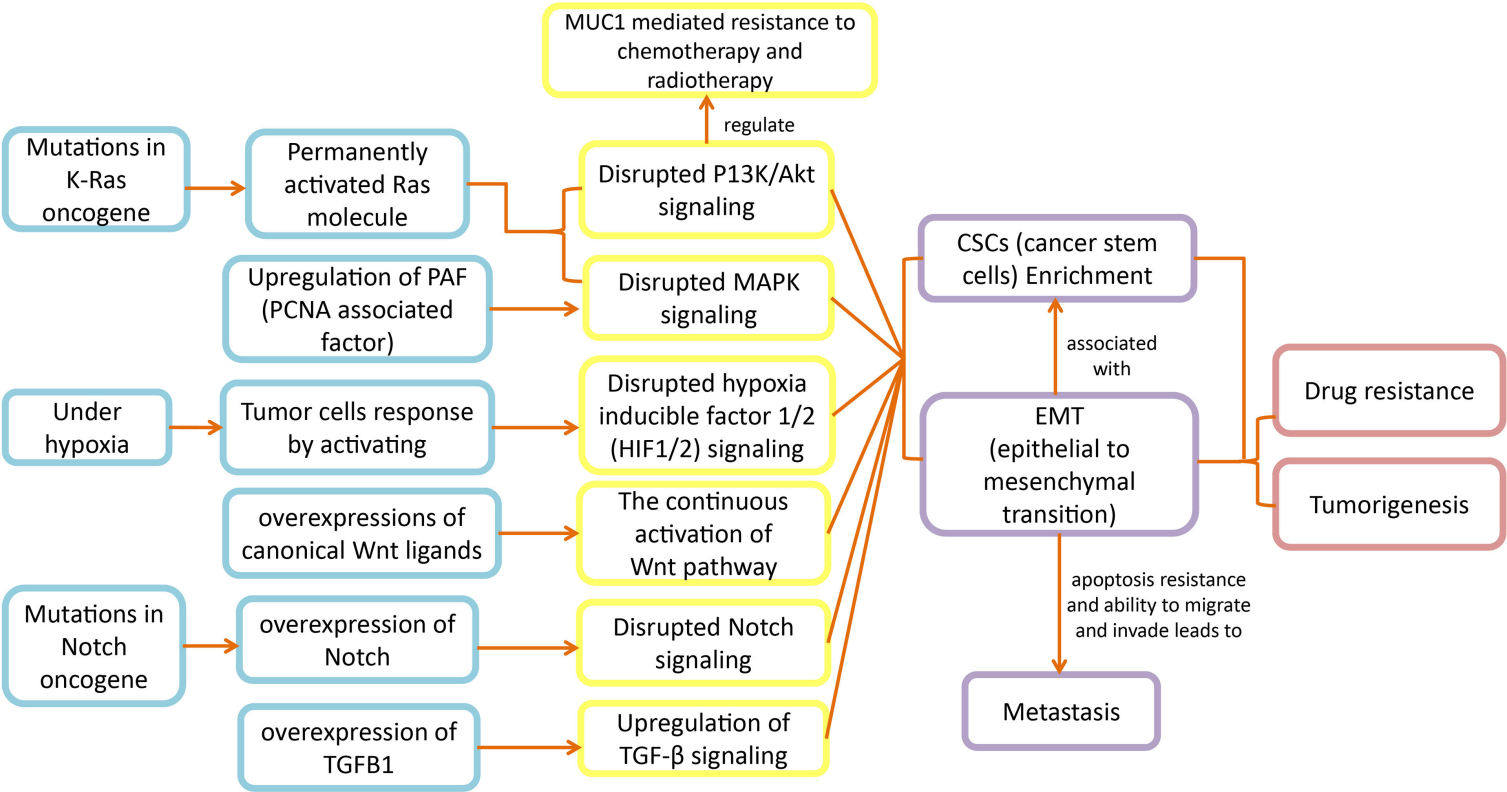
Disrupted pathways in pancreatic cancer: Disruptions in K-Ras, hypoxia, Notch signaling, MAPK, PI3K/Akt and TGF-β signaling pathways lead to EMT and CSCs development, which ultimately expedite metastasis and chemoresistance in pancreatic cancer patients.

Disturbances in signaling pathways cause numerous disease states including malignancies (36, 37). Signaling pathways, a complex network of cytokines, transcription factors, and the tumor microenvironment are responsible to regulate EMT that exhibits CSC-like properties, depicted in (**Figure 1**) (15). The EMT of solid cancer (breast or prostate) cells results in increased metastasis by elevating the migratory and invading properties of these cancer cells. Even though in pancreatic cancer the outcome of EMT on cancer cells and clinical therapy is still debatable (15, 16), the therapeutic approach of combining chemotherapy with EMT inhibition still seems promising (15). Additionally, targeting the interactivities of inflammation and EMT by anti-inflammatory therapy is proved to be an effective approach for dealing with premalignant tumor development (15).

The cancer cells acquire motility and invading properties after the initiation of EMT, which leads cancerous cells to metastasize. Further, the EMT transcription factors also aid cancerous cells in gaining stem cell like properties. Hence, when these cancerous mesenchymal cells reach their destination (metastatic sites), they go through mesenchymal-epithelial transition (MET), to attain back the epithelial phenotype. The MET results in cancerous cells colonization in distant locations (16, 24). The apoptosis resistance and motility properties make EMT an important factor to consider in carcinoma development (38, 39). The pathological analyses have revealed the EMT associated molecules in surgically resected pancreatic cancer samples (40) and in mouse models exhibiting invasive pancreatic cancer (41). Based on these analyses, EMT could be proposed as a significant biochemical mechanism in the progression of pancreatic cancer.

#### 2.2.1 K-Ras

K-Ras stands for ‘Kirsten RAS oncogene homolog from the mammalian RAS gene family. It is an oncogene that encodes the respective K-Ras protein. K-Ras protein is a small GTPase transductor protein that functions in the regulation of cell division by transmitting the external signals to the nucleus of the cell (42). The K-Ras protein becomes activated after the activation of tyrosine kinases, and with the epidermal growth factor (EGF) binding to its receptor, known as epidermal growth factor receptor (EGFR) (42). In the activated form K-Ras binds to GTP and transfers (transduce) the activation signal to the nucleus of the cell through MAPK and P13K/Akt led cascades, hereby regulating cell transformation (42). Alterations in the oncogenic K-Ras gene result in loss of K-Ras protein’s capability to transform between active and inactive states (42), as RAS molecules become permanently activated due to inhibition of GTP hydrolysis (43). These mutations in K-Ras eventually cause resistance to chemotherapy, also including those therapies that particularly target epidermal growth factor receptors (42). Point mutations in the K-Ras gene are observed in various human tumors (44), including pancreatic carcinomas (42). Fascinatingly, at the time of diagnosis, the occurrence of mutations in the K-Ras gene is highly observed (> 80% of cases) in patients suffering from pancreatic cancer (45). Alterations in KRAS are responsible for setting up genetic events that lead normal pancreatic tissue to PDAC, and this transition includes sophisticated steps of genetic changes that last around 12 years (46). KRAS is one of the most frequently mutated genes in cancers (46–48) and its mutations are observed in more than 90% of PDAC patients involved in tumor formation and development (47). In pancreatic cancer, oncogenic K-Ras is known to stimulate multiple signaling pathways that are related to cancer cell survival. For this reason, K-Ras signaling could be taken as a perfect target in pancreatic cancer to offset the cancer continuation (49). KRAS is proposed as a significant therapeutic target, however, designing inhibitors against this potent therapeutic is challenging (47, 50). The absence of binding pockets for drugs on KRAS protein, makes inhibitor designing a challenge (48, 51). Though KRAS protein has a nucleotide binding site, still targeting this would also be difficult because of the high affinity of KRAS for GDP and GTP (48). Hence, most of the research in the past on targeting KRAS is focused on indirect approaches (46). Various drug designing strategies aiming at indirect targeting have been failed in the past (47, 50). Because of that, direct targeting of RAS genes is reckoned feasible (50). Lately, a new approach of direct covalent targeting of G12C mutant KRAS has revealed a potential dynamic binding pocket (48, 51). Based on this discovery, numerous structure based drug designing projects to directly inhibit KRAS are initiated (48).

#### 2.2.2 P13K Signaling Pathway

The altered KRAS modulates many signaling pathways including phosphatidylinositol 3-kinase (PI3K) signaling. Disruptions in P13K signaling and its subsequent downstream signaling components significantly regulate various oncogenes that are involved in various cancers, including PDAC (52). Apart from KRAS dependent regulation, P13K signaling cascade activation is associated with growth factor stimuli and cytokines. Cytokines enter the cancer cells through high affinity cell surface receptors, known as receptor tyrosine kinases (RTKs). After initiation, P13K downstream signaling mediates various oncogenic functions like cancer cell metabolism, growth, and movement by further activating other signaling pathways (52).

The P13K signaling pathway substantially contributes to the progression of pancreatic cancer (49) as the P13K/Akt is activated in both PDAC and K-Ras drove pancreatic cancer mouse models (53, 54). Moreover, P13k signaling might also control MUC1 mediated chemotherapy and radiotherapy resistance (55). MUC1 significantly contributes to pancreatic cancer development as it modulates the multidrug resistance genes’ expressions through both Akt-dependent and independent pathways (56). Consequently, the regulatory axis of MUC1-P13k signaling might be suggested as a potent therapeutic target in pancreatic cancer (49).

In some cancers (breast and ovarian non-small cell lung cancer (NSLC), inhibition of P13K signaling nodes has revealed promising outcomes. However, in PDAC, inhibition of the P13K cascade by using monotherapies of these same drugs (small molecule inhibitors), has not resulted in a favorable therapeutic effect (52). In recent times, the focus has been shifted to a combinatorial regimen rather than relying on monotherapy. To efficiently thwart the tumor development in PDAC, P13K inhibition along with its downstream pathway is suggested by using P13K specific inhibitors and molecule attenuators of downstream signaling (52).

#### 2.2.3 MAPK Signaling Pathway

It is evident from the literature that the mitogen-activated protein kinase (MAPK) signaling pathway is disrupted in various diseases including cancers (57). MAPKs are serine-threonine kinases that are involved in intracellular signaling related to various cellular activities like proliferation, differentiation, transformation, and apoptosis (58, 59). The mammalian MAPKs include ERK (extracellular signal-regulated kinase), JNK (c-Jun NH2-terminal kinase), and p38 (57). The disruption of MAPK signaling also markedly contribute to the progression of pancreatic cancer. (60) explored the specific function of PAF (PCNA associated factor) in regulating MAPK signaling and reported the upregulation of PAF and its significant function in controlling the proliferation of pancreatic cancer cells. Alterations in certain oncogenes (KRAS or BRAF) and remarkable underexpression of DUSP6 play a role in MAPK activation (61). The MAPK activation contributes to the pathogenesis of pancreatic cancer by further initiating the expressions of disease related genes. The targeting of these downstream genes of MAPK signaling might result in therapeutic effects in pancreatic cancer (61).

#### 2.2.4 TGF-*β* Signaling Pathway

Transforming growth factor-B (TGF-*β*) includes a family of structurally comparable proteins. These proteins are, TGF-*β*, bone morphogenic proteins (BMPs), and activins/inhibins (62). The various important cellular functions like migration, differentiation, proliferation, apoptosis, and EMT are regulated by TGF-*β* signaling (62, 63). The dysregulation in TGF-*β* signaling is associated with cancers (62). Fascinatingly, TGF-*β* displays a dual function by showing both the tumor suppressive properties during the initial stages of cancer, and the tumor promoting properties during later cancer stages (62, 63). The tumor suppressive properties are indicated by preventing cell cycle progression and apoptosis promotion, while the tumor promoting properties are expressed by increased metastasis (62). Moreover, TGF- regulates other cell functions by either acting synergistically or antagonistically with other signaling pathways (62). Therapies targeting TGF-*β* have shown promising results in inhibition of metastasis in preclinical trials by restricting cancer cell mobility and invasion. Nevertheless, the expected favorable outcomes are not attained when these therapies were used in clinical trials. But the anticancer activity of these TGF-*β* targeting drugs improved when administrated in combination with immune checkpoint inhibitors (64).

The deregulation of TGF-*β* signaling is also implicated with pancreatic cancer (63, 65). The TGF-*β* is observed to be upregulated in pancreatic cancer, and this upregulation is implicated with venous invasion, disease progression, advanced tumor stages, liver metastasis, and eventually poor survival rate (66–70). TGF-*β* exhibits a dual function in pancreatic cancer as well by revealing both the tumor suppressor and tumor promoter properties in the initial and later stages of pancreatic cancer, respectively (71). Hence, in pancreatic cancer TGF-*β* exhibits a dual role based on cancer stages and microenvironment. The changes in TGF-*β* components are widely prevalent in pancreatic cancer and are considered to be related to metastasis (63). Further, TGF-*β* fundamentally contributes to the tumor microenvironment and CSCs in pancreatic cancer. Consequently, numerous studies demonstrating the targeting of TGF-*β* signaling have revealed promising outcomes in pancreatic cancer by showing reduced metastasis and cancer cell growth (72–76). TGF-*β* targeting should be further explored for improved treatment of pancreatic cancer (63).

#### 2.2.5 Hypoxia Signaling Pathway

Hypoxia refers to conditions of poor oxygenation. It is observed in various solid tumors. Treatment resistance and biological changes mediated by hypoxia result in an increased rate of metastasis. Under hypoxia, tumor cells react by stimulating certain signaling pathways that are oxygen sensitive. These pathways are hypoxia inducible factor 1/2 (HIF1/2) signaling pathways and the unfolded protein response (UPR). The alterations of these signaling pathways result in disrupted gene expression that enables tumor cells to survive under hypoxia (77). Changes in the hypoxia signaling cascade result in neovascularization that eventually leads to tumor invasion (78). The significant contribution of HIF in tumor development is well understood as studies have revealed the overexpression of HIF-1a and HIF-2a in metastatic cancers of humans, and this overexpression corresponds to tumor angiogenesis and mortality rate of patients (79, 80).

Hypoxic regions are the characteristic feature of pancreatic cancer (81). It is highly considered to relate to both the poor prognosis and pancreatic cancer development (81). In pancreatic cancer hypoxia causes EMT, thus promoting metastasis and also reducing the effect of chemo and radiotherapies (82). Hypoxia is proposed as a potential therapeutic target for a highly fatal malignancy of pancreatic cancer (82).

#### 2.2.6 Wnt Signaling Pathway

Wnt signaling substantially contributes to embryonic development and normal adult homeostasis (83). Int-1 belongs to the Wnt gene family and was discovered as a proto-oncogene in mice. Though, five years later, Int-1 was reported as the homolog of one of the regulators of Drosophila melanogaster segment polarity, called the ‘wingless’ gene. The Wnt gene got its name by the fusion of these two genes (wingless and Int) (83). There are 19 cysteine-rich glycoproteins in the human Wnt family that act as ligands for different receptors or co-receptors (84). The Wnt signaling pathway is an evolutionary conserved regulatory pathway, further subdivided into three pathways. These sub-pathways are named as non-canonical planar cell polarity (PCP) pathway, non-canonical Wnt/calcium pathway, and canonical pathway (83). This pathway mainly functions in cell proliferation, differentiation, and survival (85). Dysregulation of this pathway is associated with various diseases, including cancers (83).

The Wnt/B-catenin pathway significantly contributes to different cells/tissues of the body. This pathway mainly functions by regulating the development of somatic stem cells in different body organs. In pancreatic cancer, this pathway facilitates carcinogenesis by regulating EMT, angiogenesis, apoptosis, stemness, and tumor microenvironment (86). This pathway has been observed to stimulate apoptosis resistance and conservation of cancer stem cells, eventually causing pathogenesis of pancreatic cancer (87). The continuous activation of the Wnt pathway and the overexpressions of canonical Wnt ligands (Wnt2, Wnt5a, and Wnt7a’s) are also observed in pancreatic cancer. Further, dysregulation of this pathway is also linked with resistance to drugs in pancreatic cancer (86).

#### 2.2.7 Notch Signaling Pathway

Notch signaling is initiated with the binding of ligands to the Notch receptor. There exist five ligands and four Notch receptors (Notch1-4) in mammals (88). Notch genes are named after the notched phenotype of Drosophila. These genes encode conserved cell surface receptors. Hence, most of the elements of the notch signaling pathway are evolutionarily conserved (89). Notch signaling significantly contributes to cell proliferation, survival, and differentiation. This pathway is reported to be frequently activated in cancers (89, 90). Notch signaling also contributes to cell motility and invading properties by initiating the expression of EMT markers, and this EMT induction is also associated with chemoresistance in cancers (91).

Notch signaling also critically functions in the progression of pancreatic cancer (92, 93). Interestingly, the dual exhibition of both the tumor suppressive and oncogenic functions by this pathway is reliant on the cellular context (92, 93). For instance, a study has reported that in skin cancer Notch-1 plays an oncosuppressive role (94). Another experimental study reported the suppression of PanIN (Pancreatic Intraepithelial Neoplasias) caused by Notch-1 in a mouse model of pancreatic cancer (95). However, various experimental studies reported that Notch plays an oncogenic role in pancreatic cancer. For instance, Notch signaling is reported to be critically involved in tumorigenesis of pancreatic cancer by promoting PanIN, which is a precursor for invasive pancreatic cancer (96). Further, numerous studies have reported the overexpression of Notch in pancreatic cells (97–101). The Notch signaling pathway’s reactivation is also related to pancreatic cancer initiation and progression, thus suggesting this pathway as a potent biomarker and therapeutic target of pancreatic cancer (92, 93).

## 3 Oncolytic Virus Therapy

Cancerous cells exhibit motile and invading abilities by escaping the immune system, thus causing harm to the body by metastasizing. To overcome this, cancer immunotherapies aid in detecting and destroying cancerous cells by boosting the immune system of the body. Fundamentally, the cancer immunotherapies disable the classical mechanism of the cancer cells by which they escape and repress the immune responses (102). Oncolytic viruses (OVs) therapy is a novel targeted immunotherapy, viruses selectively exterminate cancer cells by lysis resulting in antitumoral immune simulation. The specificity and efficiency of oncolytic viruses make it an appealing therapeutic approach. Various oncolytic DNA and RNA viruses are currently being investigated and employed for the treatment of different types of cancers. These viruses are native or genetically altered to selectively infect cancer cells (103, 104).

The first oncolytic virus that the US Food and Drug Administration has approved for treating advanced melanoma is a Herpes simplex virus, called T-VEC (105). Granulocyte-macrophage-colony-stimulating factor (GM CSF) gene is genetically incorporated into the T-VEC virus (106). H101 is an adenovirus that is genetically modified, and in China, it has been permitted to use for treating head and neck cancer (105, 107). These two approved oncolytic viruses give insight for exploring and developing new viruses and require an in-depth study of path dynamics involved in the innate and adaptive immune response against tumor cells and viruses.

### 3.1 Disrupted Signaling Pathways and Oncolytic Viruses Therapy

**Figure 2:**
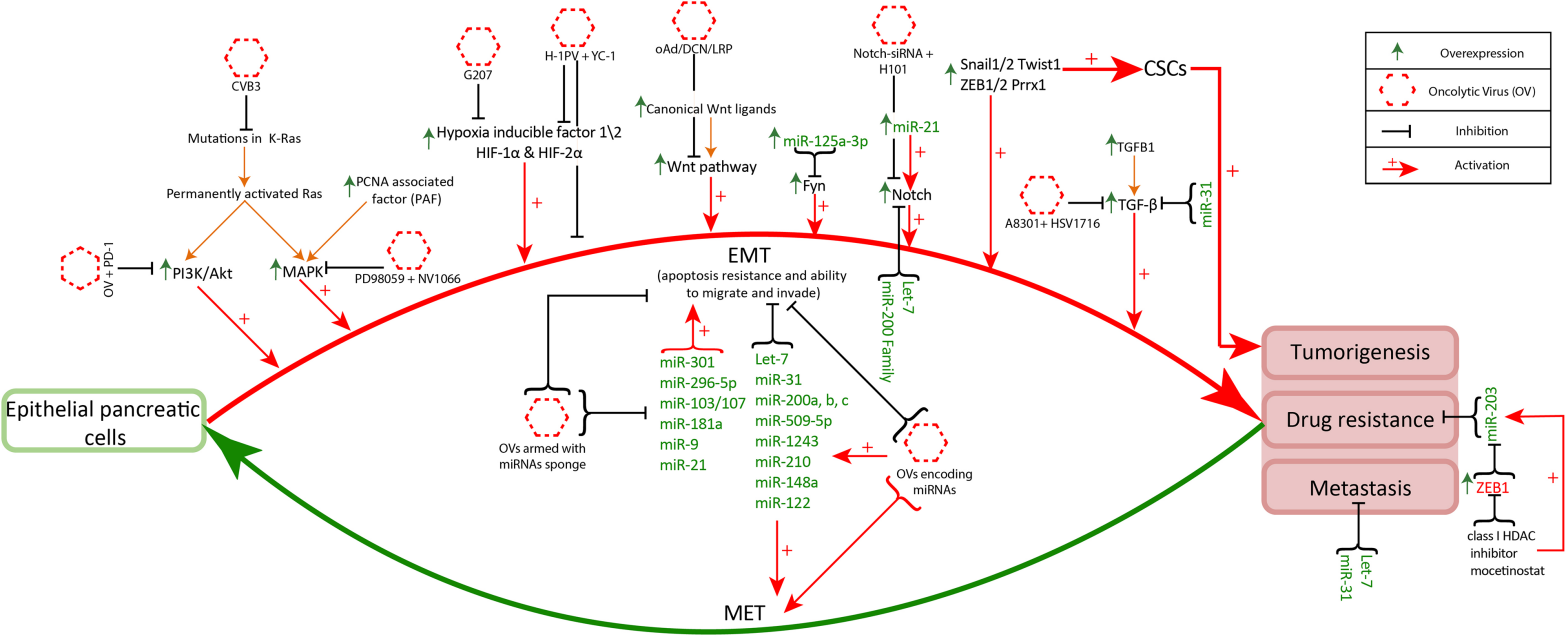
Disrupted pathways targeting with oncolytic viruses: Disruptions in K-Ras, hypoxia, Notch signaling, MAPK, PI3K/Akt and TGF-β signaling pathways facilitates increased anticancer specificity of oncolytic viruses. CVB3 and reovirus effectively infect cancer cells with mutant K-Ras (111). The administration of oncolytic viruses along with PD-1 in PI3k/Akt upregulated cancer cells can exhibit increased antitumor immune memory (112). Likewise, oncolytic viruses administration along with the inhibitors of overexpressed genes (MAPK, Wnt, TGF-β, Notch1, HIF-1α) facilitates oncolytic virus infection and enhances its antitumor property (113–116). The multiple overexpressed miRNAs (miR-301, miR-296-5p, miR-103/107, miR-181a, miR-9, miR-21) promote EMT related signaling increasing invasiveness and chemoresistance of pancreatic cancer. Targeting these miRNAs with oncolytic viruses armed with miRNAs sponges helps in the reduction of cancer progression and increases antiviral activity (117). On the other hand miRNAs (Let-7, miR-31, miR-200 family, miR-509-5p, miR-1243, miR-210, miR-148a, miR-122) regulate normal functioning in the pancreas and inhibit EMT associated signaling. These miRNAs are underexpressed in pancreatic cancer and the oncolytic viruses encoding these miRNAs have better cancer cell specificity and anticancer response (118).

The dysregulation in signaling pathways is associated with resistance to conventional therapies (chemo and radiotherapies) and metastases (108). However, this disrupted signaling of tumor cells is responsible for safety guarantees in oncolytic virus therapy. As the disruptions in signaling pathways play a critical role in the alteration of genetics and physiology of tumor cells. Thus, make viruses selective towards aberrant behaving tumor cells (109). Additionally, the function inability due to the mutations in main protein coding genes of antiviral signaling remarkably contributes to virus replication in tumor cells (110). The disrupted signaling is also inhibited to ameliorate the effect of oncolytic virus therapy. Numerous experimental studies are conducted that combined the inhibitors to selective signaling pathways with oncolytic viruses to increase the cytotoxicity of viruses for promising results in cancer treatment. Some of these studies are stated below and **Figure 2** demonstrate the targeting of disrupted pathways with oncolytic viruses for selectivity and efficacy.

Increased levels of K-Ras, which is the characteristic of tumor cells, play a role in the enhanced oncolytic capacity of Bovine Herpesvirus 1 (BHV-1) against lung cancer (119). K-Ras is targeted in K-Ras mutant lung adenocarcinoma by proposing coxsackievirus type B3 (CVB3) as a potent oncolytic agent. (120) showed the selective infection and lyses of K-Ras mutant lung adenocarcinoma cells, along with displaying negligible harm to normal cells, by CVB3 in cultured cells. The upsurge of K-Ras also facilitates the tumor selective infection of reovirus (111).

The target inhibition of PI3Kδ before the intravenous administration of vaccinia virus significantly promoted the antitumor response in immunocompetent mice (121). Further (112), experimentally demonstrated that the use of oncolytic virus along with P13K inhibition and anti-programmed cell death protein 1 (PD-1) treatment resulted in increased antitumor immune memory in glioblastoma mice model. Similarly, the decreased MAPK signaling is associated with effective treatment of triple negative breast cancers (TBNCs) by oncolytic virus therapy. (114) demonstrated that inhibition of MAPK by PD98059 resulted in synergistic outcomes of oncolytic virus NV1066, a replication-competent herpes virus, in TBNCs cell lines. The blockade of TGF-*β* by A8301 (inhibitor) resulted in improved efficacy of oncolytic herpes virus HSV1716 in murine models. (113) showed that survival time significantly increased with the combination therapy of oncolytic virus and TGF-*β* inhibitor as compared to the treatment with oncolytic virus alone. Further (122), also experimentally demonstrated that the inhibition of TGF-*β* increases the oncolytic capacity (antitumor effect) of the herpes simplex virus in glioblastoma models.

Oncolytic viruses have varied adaptabilities under hypoxia in cancerous cells, as herpes simplex viruses showed elevated replication compared to adenoviruses, while others exhibited unaltered behavior (123). In multiple cancers (77) including pancreatic cancer (82), hypoxia is associated with metastases and therapy resistance. However, this lethal hypoxic characteristic in cancer might be beneficial in oncolytic virus therapy. (124) evaluated the effect of ribonucleotide reductase (RR) enzyme produced under hypoxia, in the functioning of a PR deficient herpes simplex oncolytic virus G207. And reported the remarkably increased cytotoxicity of G207 under hypoxia induced PR, thus suggesting this oncolytic virus as a promising treatment in colorectal cancer. In another study (116), reported that H-1 oncolytic parvovirus along with HIF-1*α* inhibitor resulted in improved antitumor response with increased apoptosis in pancreatic cancer.

Wnt signaling is mostly overexpressed in colorectal cancers elevated Wnt/b-catenin signaling. (126) genetically engineered oncolytic adenovirus by inserting tumor suppressing gene (TSLC1) that specifically targets Wnt signaling. In hepatocellular carcinoma (HCC) models this oncolytic virus showed metastasis inhibition by limiting cancer cell proliferation. (115) evaluated the combined effect of recombinant oncolytic adenovirus (H101) with Notch1-siRNA. This combined therapy resulted in apoptosis of cancer cells due to Notch inhibition, along with increased cytotoxicity of H101. In a study (127), evaluated the impact of the oncolytic virus on notch signaling in tumors found in the nervous system. They reported therapeutic improvement in glioblastoma when wildtype oncolytic herpes simplex virus (HSV-1) is combined with a notch inhibitor (gamma secretase inhibitor).

Oncolytic viruses armed with miR-125a-3p, miR-216a, or miR-148a improve tumor-selectivity to replication-competent viruses, improve their safety profile, and can display strong anticancer efficacy (128, 129). (118) designed miR-122, miR-7, and miR-148a coding measles virus MV-EGFPmtd, which exhibit high pancreatic cancer cell specific targeting. Apart from the various miRNAs facilitating cancer growth, a few miRNAs also hinder the infection rate of oncolytic viruses e.g overexpressed miR-222 exhibit antiviral activity (130). The possible solution is to design oncolytic viruses armed with miRNA sponges having binding sites for these miRNAs, reducing the titer of overexpressed miRNAs and enhancing the anticancer function of viruses. A similar oncolytic virus is designed by (117) adenovirus AdNuPAR-E-miR222-S having a miR-222 binding site.

### 3.2 Preclinical and Clinical Trials of Oncolytic Viruses Therapy

#### 3.2.1 Adenovirus

Adenovirus is a member of the adenoviridae family, containing a double-stranded DNA genome of about 35 KB length (104, 106). Adenovirus is non-enveloped and has an icosahedral capsid, there are 55 serotypes of human adenovirus identified right now (131). Virus size ranges from 70 to 100 nm in diameter (131). Adenovirus enters the cell through coxsackie and adenovirus receptor (CAR) (131). Multiple genetically engineered adenoviruses are designed to improve antitumor efficacy and cancer cell selectivity. Different engineered adenoviruses include H101, Onyx-015, DNX-2401, VCN-01, AdV-tk, ad5-DS, LOAd703, CAdVEC, Colo-Ad1, ProstAtak, AdNuPARmE1A and CG0070 (106, 132–134). Pancreatic cancer is a cold tumor with an immunosuppressive tumor microenvironment (TME), so researchers are putting efforts to overcome this. Numerous preclinical studies strived to improve the effectiveness of oncolytic adenoviruses and displayed promising results. The 62% mouse model injected with Ad5/3-E2F-d24-vIL2 demonstrated a long-term survival rate along with activation of the immune response. The genetically engineered adenovirus Ad5/3-E2F-d24-vIL2 code for vIL-2, a variant of IL-2 immunomodulatory protein,and Ad5 with the Fiber knob of Ad3 (135).

(136) designed (OAds) a novel hybrid adenovirus acquired from the multiple strains (1,2,5, and 6) of serotype C. They armed OAds with RNA interference inhibitor P19, which showed a better anticancer response than Ad5 and H101. The main setback in using adenovirus as an oncolytic virus is that most of the patients have circulating anti-adenovirus neutralizing antibodies (nAbs). This hinders the evasion and antitumor activity of adenovirus. (137) designed PEGylated oligopeptide-modified poly(*β*-amino ester)s (OM-pBAEs) coated adenovirus AdNuPARmE1A. The toxicity profile and pharmacodynamics of the virus were assessed in cell lines and mouse models. AdNuPARmE1A virus not only avoids nAbs production but also effectively targets tumor cells (137). The overexpressed miR-222 in pancreatic cancer cells exhibits antiviral activity and hinders the viral cytotoxic effect. (117) designed oncolytic adenovirus AdNuPAR-E-miR222-S using AdNuPARmE1A having a miR-222 binding site to decrease the level of overexpressed miR-222 in cancer cells. They have performed *in-vitro* and *in-vivo* testing to evaluate the efficacy of the designed adenovirus. The cell lines (PANC-1 and MIA PaCa-2) testing signifies the 1.5 fold increased release of virions particles and elevated viral cytotoxicity. The single dose of intravenously administered AdNuPAR-E-miR222-S to a mouse model implanted with PANC-1 tumor cells not only reduced the tumor size but also controlled progression for a long time (117).

To enhance the systemic delivery of oncolytic adenovirus (138) devised a novel approach of administrating human bone-marrow mesenchymal stromal cell (hMSCs) carrying replicating oncolytic adenovirus (oAd/RLX-PCDP). They had genetically engineered oAd to express relaxin (RLX) and complexed with (poly (ethyleneimine)-conjugated poly(CBA-DAH); PCDP) viral coat. *In-vivo* testing indicates RLX expression helps in degrading dense extracellular matrix (ECM) in the pancreatic cancer microenvironment TME and viral coat enhance the efficacy of systemic delivery (138). As the pancreatic cancer TME has the dense desmoplastic deposition of ECM which thwarts the chemotherapy penetration in the tumor and also makes it highly immunosuppressive. Researchers are trying to overcome this by designing recombinant adenovirus. (139) generated TNF-*α* and IL-2 expressing adenovirus (oAd-TNFa-IL2) and tested it in combination with meso-CAR T cells. The combined therapies showed an improved immune response even in immunocompetent mouse models and inhibition towards metastasis development (139). In another study relaxin (YDC002) expressing adenovirus was analyzed to overcome the ECM related chemoresistance of pancreatic TME. In a mouse model study, they have determined that the very low dose of Gemcitabine (0.01-0.05*µ* M) in combination with YDC002 showed a significant antitumor effect. The large quantity of (1-50*µ* M) Gemcitabine alone has a very low antitumor effect than the combination therapy (140). Moreover (141), used disrupted Wnt signaling and target ECM deposition by designing oncolytic adenovirus (oAd/DCN/LRP). For effective systemic administration, they cover the viral coat with neurotensin peptide (NT)-conjugated polyethylene glycol (PEG) oAd/DCN/LRP-PEG-NT.

(142) developed a genetically engineered adenovirus (ICOVIR15) coding for miR99b and miR485, for facilitating the viral propagation and escalating the antitumoral activity in the pancreatic cancer cells. The Interferon-α (IFN*α* ) in combination with chemoradiation therapy shows an improved survival rate along with the systemic toxicity in pancreatic cancer patients. (143) devised IFN*α* expressing adenovirus OAd-hamIFN to reduce systemic toxicity and increase the intratumor level of cytokine. They also assert the improved efficacy of OAd-hamIFN in combination with chemoradiation on the immunocompetent hamster model (143). A preclinical study (144) designed Ad5-3Δ -A20T mutated adenovirus which can effectively infect and replicate in *α* v*β* 6 integrin expressing cells. They checked the efficacy of Ad5-3Δ -A20T alone and along with Gemcitabine on pancreatic cancer *in-vivo* model. In both scenarios, Ad5-3Δ -A20T showed high selectivity to pancreatic cancer cells (144). In a preclinical study (145), devise that overexpression of PKM2 results in tumor progression and aggressiveness of pancreatic cancer. They constructed a PKM2 inhibiting adenovirus (OAd.R.shPKM2). In pancreatic cancer xenograft model testing of OAd.R.shPKM2, the virus induces apoptosis and a strong antitumor effect.

In a preclinical study (146), designed recombinant adenovirus (ZD55−TRAIL-IETD-Smac) expressing TRAIL and Smac gene. They investigated the synergistic effect of SNS-032 (CDK inhibitor) in combination with ZD55−TRAIL-IETD-Smac in a pancreatic cancer xenograft model. They determined that SNS-032 enhances the antitumoral effect of ZD55-TRAIL-IETD−Smac (146). A preclinical study (147) checked the efficacy of Delta-24-RGD (DNX-2401) adenovirus against pancreatic cancer. Delta-24-RGD easily replicates in cells with defective P16/RB/E2F pathway and has already shown promising results against brain cancer in phase I clinical trial (148). Delta-24-RGD also inhibits the progression of tumor cells in pancreatic cancer cell lines. In a preclinical study (149), proposed a mechanism to treat Gemcitabine resistant pancreatic cancer. Survivin hinders the activity of Gemcitabine, they constructed an shRNA-encoding adenovirus which inhibits survivin. The combination of shRNA-encoding adenovirus, Gemcitabine, and TRAIL increase the cytotoxic effect of Gemcitabine. Overall this combination causes tumor size regression. In a study (150), compared the efficacy of two recombinant adenoviruses (OAV, HDAd) encoding IL-12 in a hamster pancreatic cancer model. The OAV caused an elevated level of IL-12 in cancer cells and causes severe toxicity. While the HDAd allows the controlled release of IL-12 from the liver and results in tumor growth inhibition (151). checked the safety profile and antitumor efficacy of VCN-01 adenovirus in pancreatic cancer mice and hamster models. Due to VCN-01 integrin binding selectivity and effective replication in the cell with disrupted pRB pathway, it shows promising results. VCN-01 is also under consideration in two clinical trials in combination with chemotherapies (**Table 1**).

**Table 1:** Clinical trials for assessing the oncolytic adenovirus and Herpes Simplex Virus therapy effect in patients suffering from pancreatic cancer.

Phase	Oncolytic virus	Site of Admin	Combined with	No of Patients	Patients’ Info	Year	mPFS (Months)	mOS (Months)	Study
**Adenovirus**									
I	AdV-tk	Intratumoral injection	Valacyclovir	27	Arm A: Resectable Arm B: unresectable LAPC	Start: 2008 End: 2015	Arm A: - Arm B: 5.8	Arm A: 11 Arm B: 12	(NCT00638612) (133)
I	(Ad5-DS)	Intratumoral injection	5- Fluorocytosine /Valganciclovir /Standard dose of intravenous Gemcitabine	9	LAPC	Start: 2016 End: 2019	11.4	–	(NCT00638612) (133)
I	(Ad5-DS)	–	5-fluorocytosine/ valganciclovir	8	Non-metastatic LAPC	Start: 2006 Terminated (Poor enrollment)	–	–	(NCT00415454)
I	CAdVEC	Intratumoral injection	–	45 Patients with 10 different malignances	LAPC	Start: 2020 End: 2038	–	–	(NCT03740256)
I, II	LOAd703	Intratumoral injection	–	50 Patients with 4 different malignances	LAPC	Start: 2018 End: 2022	–	–	(NCT03225989)
**Herpes Simplex Virus**									
I	HF10 (HSV)	Intratumoral injection	Erlotinib/Gemcitabine	10	Unresectable LAPC	Study End: 2018	6.3	15.5	(152)
I, II	OH2	Intratumoral injection	–	25	LAPC or metastatic	Start: 2021 End: 2022	–	–	(NCT04637698)
I	T-VEC	Intratumoral injection	–	16	LAPC or metastatic	Start: 2017 End: 2026	–	–	(NCT03086642) (NCT03252808)
I	HF10 (HSV)	Intratumoral injection	Gemcitabine/ Nab-paclitaxel/ TS-1	36	LAPC and never received anticancer therapy	Start: 2017 End: 2035	–	–	(153)
I	HF10 (HSV)	Intratumoral injection	–	Total: 17 8 pancreatic cancer patients	Unresectable LAPC	Start: 2006 End: 2014	–	–	(154)
I	HF10 (HSV)	Intratumoral injection	–	Total: 9 3 pancreatic cancer patients	LAPC and metastatic	Start: 2005 End: 2007	–	–	(155)

Several clinical trials are going on to check the efficacy of oncolytic adenoviruses against pancreatic cancer. These clinical trials include recombinant and native adenoviruses injected separated or along with other therapies to the patients. A phase I, II clinical trial uses the intratumoral injection of ONYX-015 (adenovirus with 827 bp deletion in E1B region) which helps in the accumulation of p53 and evokes apoptosis. They checked the effectiveness of ONYX-015 in combination with Gemcitabine in 21 LAPC patients. The study was completed in the year 2003, results are published (156). In another phase I clinical trial intratumoral injection of ONYX-015 was administrated to 23 LAPC patients. The study was completed in the year 2001, published as (132). A phase I clinical trial checked the efficacy of AdV-tk along with Valacyclovir (antiherpetic prodrug). AdV-tk is a recombinant adenovirus encoding herpes simplex virus thymidine kinase (157). The intratumoral injection of AdV-tk is given to 27 patients divided into two groups including resectable tumor and unresectable LAPC. The study (NCT00638612) was completed in the year 2015, results are published as (133). A phase I clinical trial study administrated Ad5-DS (Ad5-yCD/mutTKSR39rep-ADP) along with chemotherapies (5-Fluorocytosine + Valacyclovir + Gemcitabine) to 9 LAPC patients. The study was completed in the year 2019, results are published (134). Out of all adenovirus trials (134) reported the longest overall progression free survival of patients, the phase II trial should be run to further evaluate the performance of this combination therapy.

A phase I and II clinical trial study administrated LOAd703 along with therapies (Gemcitabine + Nab-paclitaxel +/- anti-PD-L1 antibody Atezolizumab) to 43 LAPC patients. The LOAd703 is a recombinant adenovirus encoding two genes TMZ-CD40L and 4-1BBL. These help in the stimulation of antitumor immune cells (macrophages, natural killer cells, CD4+ and CD8+ T cells). The estimated date of study (NCT02705196) completion is Dec 2021. Two phase I clinical trial studies utilizes VCN-01 adenovirus. They give the intravenous and intratumoral injection in combination with Abraxane/Gemcitabine to LAPC. The trial (NCT02045589) was completed in the year 2018, while the study (NCT02045602) completion date was the year 2020, results for both studies were not published. Another phase I clinical study checked the impact of Ad5-DS along with 5-fluorocytosine (5-FC) and valganciclovir (vGCV) in 8 non-metastatic LAPC patients. The study (NCT00415454) was terminated due to the poor enrollment of patients. A phase I, II clinical trial study is running to check the effectiveness of intratumoral injection of LOAd703 in 4 different malignancies. They (NCT03225989) also enrolled LAPC patients study will complete in the year 2022. In a phase I clinical trial immunomodulatory molecule expressing adenovirus (CAdVEC) is considered for 10 different malignancies, including LAPC patients. They (NCT03740256) are administrating CAdVEC through intratumoral injection alone or in combination with (HER2 specific CART cells) to the patients. The expected date of study completion is the year 2038. A total of 10 clinical studies were identified, the information of 5 clinical trials is provided in **Table 1**, other 5 studies were previously cited in (158).

#### 3.2.2 Herpes Simplex Virus

Herpes virus is a double-stranded DNA virus with a genome length of 154 KB, it belongs to the herpesviridae family (103). It is enveloped virus with an icosahedral capsid and has a size of 200nm approximately. Herpes virus enters the cell using herpesvirus entry mediator (HVEM), nectin1, and nectin2 (103, 159). Genetically engineered herpes viruses used in different clinical trials are T-VEC, G207, HF10, HSV1716, and OrienX010 (103). Virulence gene is deleted in all attenuated viruses, while granulocyte-macrophage colony-stimulating factor (GM-CSF) gene is introduced in Talimogene laherparepvec (T-VEC) and OrienX010 (160). The Canerpaturev (C-REV) is a mutated herpes simplex virus with lacking expression of UL43, UL49.5, UL55, UL56, and (LAT).15. The absence of UL56 and (LAT).15 results in depletion of viral pathogenicity and neuroinvasion making C-REV safe to use as an oncolytic virus (161, 162).. CD8+ T cell activity plays a fundamental role in the efficacy of oncolytic virus therapy. (PD-1/PD-L1) overexpression hinders the activation of CD8+ T cells, a high level of PD-L1 is also reported in pancreatic cancer cells. *In-vitro* and *in-vivo* testing of C-REV displayed effective tumor regression even with the high PD-L1 expression (163).

In a preclinical trial (164), checked the effect of (oHSV) herpes simplex virus-1 on the immunocompetent pancreatic cancer model. It not only increases the activity of antitumor immune cells (CD8+ T and CD4+ T cells) but also reduces the immune suppressor macrophages. The oHSV displayed the effective reduction of tumor size along with progression-free survival in immunocompetent mice (164). (165) investigated the effect of intratumoral dissemination of oHSV-CD40L in mouse model. The oHSV-CD40L is herpes simplex virus-1 (oHSV) armed with CD40L (CD40 ligand). CD40L helps in the activation of antigen presenting cells APCs. They have identified the increase of APCs like dendritic cells (DCs) in TME and DCs mediates the activation of CD4+ T and CD8+ T cells. The activated immune response in the mouse model resulted in prolonged survival. In two studies (166, 167) checked the effectiveness of HF10 separated and along with erlotinib on xenograft model incorporating pancreatic cancer cell lines BxPC-3 and PANC-1. They determined that HF10 displayed significant results in both cases. In a preclinical trial (168) analyze the Myb34.5 herpes simplex virus type 1 (HSV-1) with mutated ICP6 gene. It displayed effective replication in pancreatic tumor cells with overexpressed B-myb gene. They also analyzed the efficacy of Myb34.5 in a mouse model along with Gemcitabine. Even the low dose of both in combination results in effective tumor size reduction (168). A preclinical study (169) found that (HSVGM-CSF) demonstrated effective antitumor activity and activated immune cells for long-term tumor control. The (HSVGM-CSF) is a recombinant herpes simplex virus with 3 defective/deleted genes (γ134.5, ICP47, and ICP6) and armed with the GM-CSF gene. In a pancreatic cancer cell line based testing (170) checked the effect of 5-fluorouracil (5-FU), Irinotecan (CPT-11), Methotrexate (MTX), or a cytokine (tumor necrosis factor-*α* (TNF-*α*)) in combination with HSV-1. They determined that MTX hinders the replication of HSV-1 in tumor cells. While 5-FU, CPT-11, and TNF-*α* improve the viral replication and increase the activity of HSV-1.

Herpes Simplex Virus shows favorable outcomes in preclinical trials for pancreatic cancer and other cancer types. Various clinical studies are accomplished and some are yet going on to analyze the upsurge in patient survival rate on the administration of oncolytic herpes virus. A Phase I, II clinical trial administrated OH2 to 21 LAPC patients through intratumoral injection. The OH2 is a recombinant herpes simplex virus with deleted ICP34.5 and ICP47 genes and armed with the human GM-CSF gene. The study (NCT04637698) expected completion date is 2022. A total of 6 different clinical trials administrated HF10 herpes virus alone or in combination with different chemotherapies (**Table 1**). A phase I clinical trial (NCT03252808), recruiting 36 LAPC patients, is expected to be completed by the year 2035. They are administrating HF10 (intratumoral injection) along with Gemcitabine+Nab-paclitaxel or TS-1 (153). Other 5 clinical trials of HF10 were completed. One study (NCT02428036) administrated (intratumoral injection) HF10 was completed in 2017, but the results were not published. In one clinical trial (phase I) intratumoral injection of HF10 was administrated to 6 LAPC or metastatic patients. The patients showed a median overall survival rate of 6.3 months (171). Another phase I study also administrated intratumoral injection of HF10 to 17 LAPC patients (154). A phase I clinical trial study administrated intratumoral injection of HF10 in combination with Erlotinib and Gemcitabine to 10 LAPC patients. The patients showed 6.3 months median progression-free survival, and 15.5 months median overall survival (152). Out of all herpes virus trials (152) reported the longest overall survival of patients, the phase II trial should be run to further evaluate the performance of this combination therapy. **Table 1** summarizes the important information from 7 clinical trial studies of the oncolytic herpes virus. Overall 11 clinical studies were identified out of which 4 studies were cited in a previous review article (158).

#### 3.2.3 Reovirus

Reovirus is a non-enveloped RNA virus, with an icosahedral capsid. It is a constituent of the Reoviridae family. It is a double-stranded RNA virus, with a genome size of 23.5 KB and its size is about 75nm (106). Reovirus is non-pathogenic to humans, normal cells of the body are resistant to reovirus attack (111). It selectively infects cancer cells and replicates due to active RAS signaling pathway, K-Ras, BRAF, and EGFR mutations in cancer cells (111, 172). The wild-type reovirus is frequently opted in numerous clinical trials for a large variety of cancers due to its efficient oncolytic nature. Its effectiveness is evaluated in the management of ovarian cancer, melanoma, colorectal cancer, multiple myeloma, head and neck cancer, glioma, non-small cell lung cancer, and myeloid leukemias (106, 173). In clinical trials, patients are checked for the molecular indicators necessary for reovirus effectiveness. Even REOLYSIN (Reovirus Type 3 Dearing) is under evaluation against metastatic or repeated Squamous Cell Carcinoma of the Head and Neck in phase III clinical trial (NCT01166542).

In a preclinical study (174), analyzed the efficacy of reovirus (Reolysin) in combination with bortezomib (BZ). The *in-vitro* and *in-vivo* testing of combination for pancreatic cancer treatment increases the apoptosis in cancer cells and shows an elevated level of anticancer response (174). (175) designed a recombinant reovirus (rS1-mmGMCSF and rS1-hsGMCSF) expressing GM-CSF gene. A herpes simplex virus, T-VEC, that expresses GM-CSF is the first oncolytic virus approved by FDA (176). The preclinical pancreatic cancer murine model testing of rS1-mmGMCSF expressing murine GM-CSF showed the systemic improvement in immune cells (DC and T cell) antitumoral activity (175). CD3-bispecific antibodies (CD3-bsAbs) is cancer immunotherapy FDA approved it for the treatment of B-cell acute lymphoblastic leukemia (ALL) in the year 2014 (177). CD3-bsAbs also exhibited favorable outcomes in pancreatic cancer preclinical studies (178). (179) uses reovirus to boost the antitumor effect of CD3-bsAbs. The mouse models were checked for the early, simultaneous, and after administration of reovirus with CD3-bsAbs. The results indicate that early administrated reovirus increases the antitumor efficacy of CD3-bsAbs and this therapy also induces the regression of non-injected distant lesions (179).

Reovirus efficacy as an oncolytic virus in combination with chemotherapies is also evaluated in 3 pancreatic cancer clinical trials. A phase II clinical trial evaluates the effectiveness of Reolysin intravenous injection along with carboplatin and paclitaxel in LAPC and metastatic patients. The study is completed in 2016 and its results are published (NCT01280058) (180). A phase II clinical trial analyzed the Reolysin intravenous injection in combination with Gemcitabine on LAPC or metastatic patients. Patients showed a median survival rate of 10.3 months, the study was completed in 2015 (NCT00998322) and results are published (181) A phase I clinical trial study of the chemotherapy along with REOLYSIN intravenous injection in advanced stage or metastatic patients. A combination of three chemotherapies comprising Gemcitabine, Pembrolizumab, and Irinotecan or Leucovorin or 5-fluorouracil (5-FU) was administrated to patients according to their condition. The study was completed in 2018 (NCT02620423) and results are published (182). **Table 2** summarizes all the important information of all 3 clinical trial studies along with their publications references.

**Table 2:** Clinical trials for assessing the effect of oncolytic reovirus, parvovirus, and vaccinia virus therapy in patients suffering from pancreatic cancer.

Phase	Oncolytic virus	Site of Admin	Combined with	No of Patients	Patients’ Info	Year	mPFS (Months)	mOS (Months)	Study
**Reovirus**									
II	REOLYSIN	intravenous injection	Carboplatin/Paclitaxel	73	Arm A: 36 Carboplatin + Paclitaxel + Reolysin Arm B: 37 Carboplatin + Paclitaxel	Start: 2010 End: 2016	Arm A: 4.9 Arm B: 5.2	Arm A: 7.3 Arm B: 8	(NCT01280058) (180)
II	REOLYSIN	Intravenous injection	Gemcitabine	34	LAPC or metastatic	Start: 2009 End: 2015	3.4	10.2	(NCT00998322) (181)
I	REOLYSIN	Intravenous injection	Gemcitabine/ Irinotecan/ Leucovorin/ 5-fluorouracil/ Pembrolizumab	11	Advanced or metastatic	Start: 2015 End: 2018	2.0	3.1	(NCT02620423) (182)
**Parvovirus**									
II	ParvOryx	Intravenous and Intratumoral injection	Gemcitabine Started after 28 days of H-1PV first dose	7	Unresectable LAPC with at least one hepatic metastasis	Start: 2015 End: 2018	3.4	5.8	(NCT02653313) (183, 184)
**Vaccinia virus**									
I	VV expressing p53	Subcutaneous injection	–	12 5 pancreatic cancer Patients 16	Unresectable and chemotherapy resistant or recurrent tumors	Start: 2011 End: 2013	–	–	(NCT01191684) (185)
I	vvDD	Intratumoral injection	–	2 Pancreatic cancer Patients	Advanced solid tumors	Study End: 2014	–	–	(186)

#### 3.2.4 Vaccinia Virus

Vaccinia Virus is a member of the poxviridae family. It is a double-stranded DNA virus, with a genome length of about 190 KB (187). Vaccinia virus is covered with a complex coat and capsid. The size of the virus ranges from 70 to 100 nm in diameter (187). To increase pancreatic cancer cell-specific lytic action recombinant vaccinia virus was designed for preclinical and clinical studies (185, 186, 188). (189) use MDRVV vaccinia virus with deleted VGF and O1 genes and recombinant MAPK gene. They fused deaminase and uracil phosphoribosyltransferase (CD/UPRT) coding genes in the MDRVV genome. The (CD/UPRT)-armed MDRVV alone and along with 5-fluorocytosine (5-FC) showed similarly improved antitumor activity in the mouse model (189).

(190) investigated the efficiency of vaccinia virus mpJX-594 (mpJX) in combination with anti-programmed death receptor-1 antibody (aPD1) in pancreatic neuroendocrine tumors (PanNETs). The aPD1 and mpJX activate the elevated antitumor immune response in mice models, with an increased level of apoptosis and reduced proliferation (191). constructed recombinant vaccinia virus VVL-21 expressing B5R and interleukin-21 (IL-21) genes to combat pancreatic cancer. They analyzed the antitumoral effect of systemically administrated VVl-21 in combination with *α* -programmed cell death protein 1 (*α* -PD1) in metastatic murine and hamster models. The combination shows promising results with the increased level of activity of immune response (CD8+ T cells, natural killer cells, and macrophages) against cancer cells (191). In a preclinical study (192), analyzed the cytotoxicity profile of recombinant SJ-815 (IFNB1 and CES2 expressing vaccinia virus) with and without irinotecan in pancreatic cancer and melanoma bearing mouse models. The combination treated pancreatic cancer mouse model displayed significant regression in tumor size (192). In a preclinical study (193), designed oVV-Smac (Smac gene expressing vaccinia virus). They checked oVV-Smac effectiveness *in-vitro* and *in-vivo* along with Gemcitabine. The co-treatment increases apoptosis and cytotoxic effect of Gemcitabine in cancer cells (193). (194) constructed recombinant vaccinia virus (VV-ING4) armed with an inhibitor of growth family member 4 (ING4) gene. They tested the efficacy of VV-ING4 alone and along with Gemcitabine in a pancreatic cancer mouse model. VV-ING4 alone shows very good results by inducing increased cytotoxicity and apoptosis in cancer cells. In co-treatment, VV-ING4 helps Gemcitabine to work more effectively as anticancer therapy (194).

In a preclinical study (195), performed pancreatic cancer cell lines based testing of GLV-168 vaccinia virus in combination with Nab-paclitaxel + Gemcitabine. The GLV-168 is armed with 3 cassettes (*β*-galactosidase, *β*-glucuronidase, and Ruc-GFP), and exhibits an effective pro-inflammatory and antitumoral active (196, 197). (198) constructed GLV-1h151 modified vaccinia virus by removing thymidine kinase. They tested the efficacy and compared the systemic and intratumor delivery of GLV-1h151 to cancer mouse model bearing (PANC-1 cell line) (198). conducted preclinical testing of GLV-1h151 in combination with radiotherapy on pancreatic cancer cell line and mouse model. They have analyzed that combination therapy increases cytotoxicity and enhances apoptosis in cancer cells (198). One of the studies suggests that differential gene analysis might fundamentally contribute to understanding the crucial genes causing resistance to any therapy in pancreatic cancer. They checked the expression profile of pancreatic cancer cell line 6 and 24 hours after being treated with GLV-1h153. They also suggest that expression analysis before preclinical trials can help in determining the efficacy of therapy based on activated genes and pathways (199).

In another preclinical study (200), constructed VVLDTK-IL-10 recombinant vaccinia virus with deleted thymidine kinase gene and IL-10 gene equipped. They checked VVLDTK-IL-10 efficacy in two pancreatic cancer mouse models, immunocompetent and pathologically aggressive models. The 87.5% immunocompetent mice exhibit total removal of the tumor with an active immune response. The pathological model treated with VVLDTK-IL-10 showed an increase in survival time of 138 days in comparison to 69 days for the mouse model treated with the non-recombinant virus (200). The (201) designed vvdd-tdTomato-hGMCSF recombinant vaccinia virus expressing granulocyte-macrophage colony-stimulating factor (GMCSF) gene to cope with immune suppression in pancreatic cancer and tdTomato fluorophore gene. They tested vvdd-tdTomato-hGMCSF on immunocompetent hamsters. GMCSF expressing vaccinia virus helps in the total clearance of subcutaneous pancreatic lesions by the activation and invasion of neutrophils and macrophages (201).

The recombinant Vaccinia virus efficacy as an oncolytic virus is also evaluated in 2 clinical trials of advanced stage solid tumor patients including pancreatic cancer. A phase I clinical trial evaluated the effectiveness of subcutaneous inoculation of vaccinia virus expressing the p53 gene in the advanced stage, in chemotherapy-resistant patients. The study is completed in 2013 (NCT01191684) and its results are published (185). A phase I clinical trial analyzed the efficacy of vaccinia virus (vvDD) intratumoral injection to advance stage solid tumor patients comprising 2 pancreatic cancer patients. The vvDD was generated by recombination of two genes cytosine deaminase and somatostatin receptor in the VGF gene deleted vaccinia virus, the study was completed in 2014, and results are published (186). The information related to both clinical trial studies is provided in **Table 2**.

#### 3.2.5 Parvovirus

Parvovirus is a member of the Parvoviridae family. It is a non-enveloped, single-stranded DNA virus, having a genome size of 5100 bases. Parvovirus is the tiniest identified virus with a diameter of 22 nm (202, 203). Toolan and co-workers were the first ones to discover this virus during the late 1950s. Rat is its natural host, but it also shows a promising rate of infection and replication in human tumor cells (202, 204). In a study (116), checked the effectiveness of oncolytic H-1 parvovirus in pancreatic cancer cells with HIF-1*α* overexpression. H-1 reduced the level of HIF-1*α* in cancer cells. They also determined that H-1 in combination with YC-1 (HIF-1*α* inhibitor) not only reduces the HIF-1 *α* level but also shows an elevated level of apoptosis and an effective antitumor effect. The H-1PV (parvovirus) decreases the level of ISGs and HERV in pancreatic cancer cells. It shutdowns cellular innate immunity and demonstrated elevated replication in cancer cells (205).

In two preclinical studies (206), and (207) tested the efficacy of H-1PV along with Gemcitabine on a pancreatic cancer mouse model. The Gemcitabine pretreated mice showed a significant reduction in tumorsize on the administration of H-1PV. (208) demonstrated that HDAC inhibitor and (VPA) in combination with H-1PV increase the viral replication and cytotoxicity in cervical and pancreatic cancer cell lines. In a preclinical study (209), checked the effect of intratumoral and intraperitoneal injection of H-1PV in mouse models bearing peritoneal metastasis. The intratumor injection results in the reduction of the tumor even on the peritoneal site. They also administrated IFNγ with H-1PV intratumoral and intraperitoneal injection. IFNγ improved the immune response in both cases. The antitumor efficacy of parvovirus vectoring cyto/chemokines (IL-2, MCP-3/CCL7 and IP-10/CXCL10) was assessed in pancreatic cancer xenograft. parvovirus armed with IL-12 shows a strong antitumor response than the other two variants (210).

A phase II clinical trial evaluates the effectiveness of intravenous and intratumoral injection of ParvOryx native parvovirus H-1 (H-1PV). The patients were also administrated with Gemcitabine dose after 28 days of the first dose of H-1PV. The inclusion criteria for this clinical trial was that all patients must have at least one hepatic metastatic lesion. The study was completed in 2018 (NCT02653313) and results are published (183, 184) (**Table 2**).

#### 3.2.6 Measles Virus

Measles virus, an enveloped RNA virus, relates to the genus Morbillivirus, which is a member of Paramyxoviridae family (211). It is a single-stranded, negative-sense RNA virus, with an approximate genome size of 16 KB (211). The diameter of the virus range from 100-200nm (106). The measles virus enters the cell through the signaling lymphocytic activation molecule (SLAM) receptor or CD46 receptor. These receptors are overexpressed in cancer cells (211, 212). Once the measles virus enters the cancer cell it starts forming virus formation machinery and causes cancer cell lysis (211). Edmonston strain of measles virus is mostly used in clinical trials, it enters the cell through CD46 receptor (106). Until now measles virus is evaluated for treating various human cancers like ovarian cancer, multiple myeloma, lymphoma, and glioma (211). Moreover, genetically modified virus expressing antibodies to a specific tumor antigen expressed in adenocarcinoma is also studied (213).

In multiple pancreatic cancer, preclinical studies recombinant measles virus in combination with Immuno/chemotherapies were analyzed. (214) constructed recombinant measles virus (MeV-CD-FmiRTS148a) armed with 5-fluorocytosine, a prodrug of 5-fluorouracil. They checked the efficacy of MeV-CD-FmiRTS148a on pancreatic cancer cell lines and intratumoral injection in a xenograft model. The treated mice showed tumor size reduction and prolonged progression-free survival. (215) analyze the combinatorial effect of a small quantity of MeV along with Gemcitabine on the pancreatic tumor mouse model and determined >50% reduction of tumor size. The combination of HDAC inhibitor with MeV was analyzed on 4 pancreatic cancer cell lines with the intention of identifying an effective epi-virotherapy (216). Approximately 30% of pancreatic cancer cells showed overexpression of the nectin-4 surface receptor. (217) tested the infection rate and antitumor effect of recombinant measles virus (rMV-SLAMblind), which showed selective targeting of nectin-4-expressing cancer cells in xenografted mice.

The miRTS for miR-122, miR-7, and miR-148a coding measles virus MV-EGFPmtd is constructed to increase vector specificity and safety (118). So, that virus will specifically replicate in malignant cells rather than normal cells without compromising oncolytic efficacy. The *in-vitro* and *in-vivo* testing confirms the highly selective pancreatic cancer cell targeting of MV-EGFPmtd. To study the dispersion of virus in the body after intratumoral injection (218) designed a sodium iodide symporter (NIS) expressing measles virus, which helps in easy CT imaging.

#### 3.2.7 Vesicular Stomatitis Virus

Vesicular Stomatitis Virus (VSV) belongs to the Rhabdoviridae family. It is enveloped, non-segmented negative-sense RNA virus, with a genome size of 11-kb. The shape of the virion is bullet like and has a size of 185 nm x 75 nm (219, 220). The VSV preferentially infects and replicates in tumor cells, because of lacking innate immunity in tumor cells. The effectiveness of VSV against numerous malignancies was illustrated by multiple preclinical studies, which is making it a promising candidate for oncolytic viruses therapy (221). Numerous pancreatic cancer based preclinical studies also checked the efficacy of VSV as an oncolytic virus. The hybrid vesicular stomatitis virus (VSV-FH) armed with F and H envelope proteins of the measles virus shows better anticancer results in preclinical trials. (222) also tested the efficacy of VSV-FH in *in-vitro* and *in-vivo* models of hepatobiliary and pancreatic cancer. VSV-FH exhibited a strong reduction of BxPC-3 pancreatic cancer cell lines *in-vitro* but the xenograft model of BxPC-3 cells was resistant. They proposed that resistant behavior *in-vivo* testing could be due to reduced tumor infiltration of the virus, as pancreatic cancer has dense desmoplastic stroma and ECM deposition. Multiple pancreatic cancer cell line and xenograft models studies were performed for VSV, few cell lines demonstrated resistance. It was proposed that VSV attachment to cancer cell surface molecule plays a key role in resistant behavior (223–227). (223) identified two natural mutations K174E and E238K in VSV glycoprotein, which is helping in the reduction of tumor resistance towards VSV. These mutations in glycoprotein are increasing the viral attachment with cancer cell surface molecules. (224) reduce the VSV resistance in HPAF-II cell lines by co-treatment with polycations and ruxolitinib. The co-treatment increases the VSV attachment to cancer cells and ruxolitinib inhibits Jak1/2 antiviral effect.

In a study (228), interrogated the VSV resistance in highly chemoresistant pancreatic cancer cell lines. They identified that upregulated interferon-stimulated genes cause VSV resistance in pancreatic cancer. The difference of IFN signaling in different pancreatic cancer cell lines also results in VSV resistance. (225) checked three recombinant VSV regulating IFN signaling in resistant cell lines. All variants displayed improved apoptotic and antitumor immune responses, while the VSV-Δ M51 variant shows a better response than other variants. (226) analyzed the effect of 16 small molecule inhibitors in combination with VSV-Δ M51 to increase VSV oncolytic therapy potential. They identify the combination of ruxolitinib or TPCA-1 (IKK-*β* inhibitor) with VSV-Δ M51 to remove the VSV resistance in pancreatic cancer cells, by downregulating IFN-I signaling. Multiple cell line-based studies checked the combination of VSV-Δ M51 along with the regulation of IFN signaling. They determined that IFN regulation/inhibition significantly contributes to the improved oncolytic potential of VSV-Δ M51 (226, 227, 229). The overexpression of mucin 1 (MUC1) in pancreatic cancer cells also resulted in VSV resistance. (230) tested 3 VSV variants (VSV, VSV-GFP, VSV-Δ M51-GFP) on 5 pancreatic cancer mouse cell lines. The cells with overexpressed MUC1 showed reduced effectiveness of all VSV variants. While VSV-Δ M51-GFP in *in-vivo* testing on xenograft model showed improved antitumor effects. They also checked the combination of VSV-Δ M51-GFP with Gemcitabine to study improved tumor-specific immunity.

#### 3.2.8 Newcastle Disease Virus

Newcastle Disease Virus (NDV), also known as avian paramyxovirus, relates to the genus Avulavirus, which is a member of the Paramyxoviridae family. NDV is enveloped RNA virus, with a helical capsid (231). It is a single-stranded, negative-sense RNA virus, having a small genome of approximately 15 KB. NDV virus size lies between 100 to 500 nm in diameter (106). NDV enters the attacked cell through direct virus endocytosis or by fusion with the plasma membrane. NDV causes infectious diseases in birds and has no pathogenic effect in humans (231). NDV selectively infects and replicates in cancer cells because type I interferon, Bcl-xL, and small GTPase RAS overexpresses in cancer cells. Bcl-xL is an antiapoptotic protein highly expressed in cancer cells and induces an immune-suppressive microenvironment (232). NDV also easily replicates in Rac1, 38Livin39, and antiapoptotic genes expressing cells (233, 234). NDV after infecting cancer cell not only actively help in the apoptosis of cancer cell, but also activate an antitumor immune response. It is used in different clinical trials for the management of kidney cancer and other malignant cancers (231). Despite its small genome, foreign genes could be introduced into it to improve the antitumor effectiveness of NDV (106, 231).

In a study (235), assessed the cytotoxic and immune response of NDV in 11 pancreatic cancer cell lines. They determined that cells expressing a high level of IFN have a reduced replication rate and cytotoxic effects of NDV. So they deduce that INF antagonist in combination with NDV could assist the oncolytic effect of NDV (235). In a preclinical study, two mouse models were infected with NDV through intravenous inoculation. The mouse model with a low level of TGF-*β* shows effective reduction of tumor and 3 months progression-free survival. Resulted inactivation of NK cell, cytotoxic, and helper T cells which leads to long-term immune activation and tumor growth suppression. While the mouse model with a very high level of TGF-*β* displayed a reduction of tumor size for a week due to NK cells but no stimulation of cytotoxic cells and helper T cells. This results in failure of tumor reduction due to antigen-specific response and also virus inhibition (236).

In another preclinical study (237), constructed 5 different recombinants NDV to find out the better option for pancreatic cancer. They designed rNDV-GFP-F0, rNDV-hIFN*β* -F0, rNDV-NS1-F0, and rNDV-F3aa, checking their efficacy in cell lines and xenograft. The intratumor injection of rNDV-F3aa to the xenograft model only displayed tumor regression and better antitumor cytotoxicity. While in the case of rNDV-hIFN*β* -F0 the higher expression of IFN hider the viral replication (237).

## 4 Discussion and Conclusion

Pancreatic cancer’s highly malignant nature requires a deep understanding of underlying mechanisms and exploring better therapeutic options. Certain significant factors like lack of early diagnosis, severe disease symptoms depicting high metastasis rate, and unsatisfactory treatment outcome contribute to poor survival rate in pancreatic cancer. Genetic alterations and overexpression of various genes in pancreatic cancer cells make it difficult to diagnose, hyper-invasive, and therapies resistant. Pancreatic tumors exhibit a high level of ECM deposition and hypoxia leading to EMT resulting in its metastatic characterizations (13, 16). Various disrupted pathways like MUC1, TGF-*β* , MAPK, PI3K/Akt, Hypoxia, Notch, and Wnt signaling in pancreatic cancer cause therapies resistance, and disease recurrence (16, 21, 22, 238). To overcome this, cancer immunotherapies helps in detecting and destroying cancerous cells by boosting the immune system of the body, and are under consideration. Survival rate and patient’s quality of life have been tremendously improved with cancer immunotherapy treatment as compared to the treatment with standard chemo and radio therapies (239). Oncolytic viruses therapy is the a novel immunotherapy, Viruses selectively target cancer cells and generate antitumoral immune response. These oncolytic viruses specifically exploit disrupted pathways. Native and recombinant oncolytic viruses alone or in combination with other therapies show promising results against different cancer types. In recombinant oncolytic viruses exogenous genes and miRNAs which are downregulated in cancer cell are usually incorporated.

**Figure 3:**
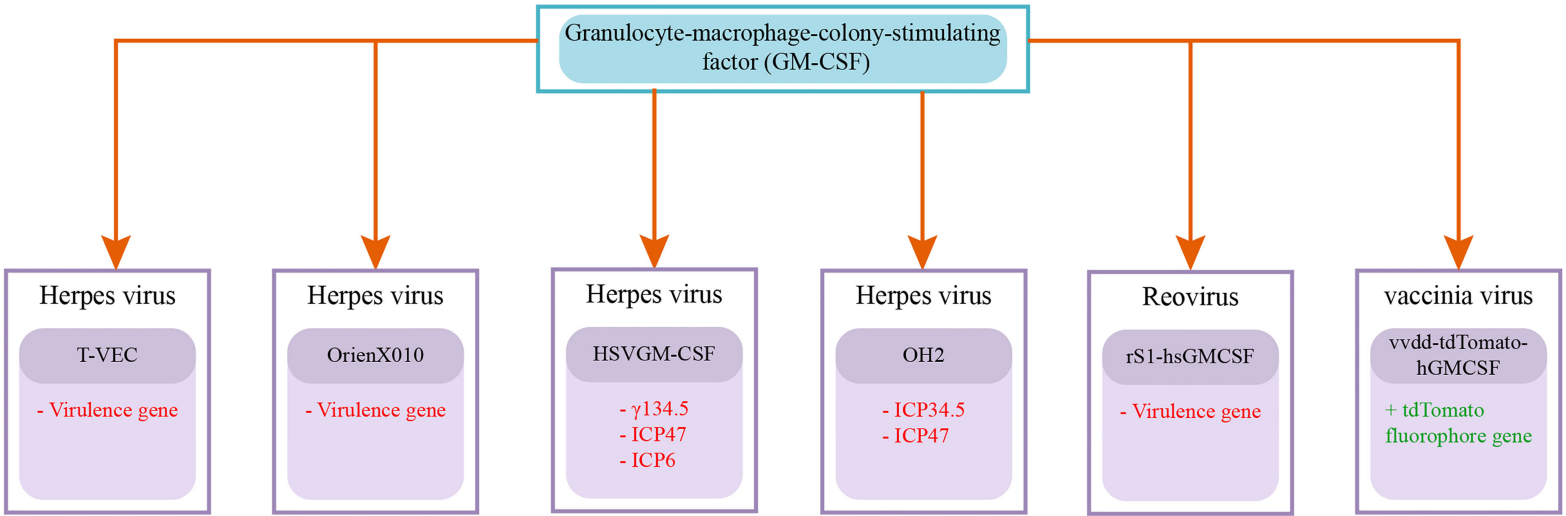
Recombinant Oncolytic Viruses armed with GM-CSF gene: GM-CSF is significant in oncolytic immunotherapy as its overexpression is associated with suppressing tumor growth (243). Talimogene laherparepvec (T-VEC) and OrienX010 are herpes simplex viruses that are genetically modified to omit and incorporate virulence and GM-CSF genes, respectively (160). HSVGM-CSF is also an oncolytic herpes simplex virus that is genetically altered to delete γ134.5, ICP47, and ICP6, and express GM-CSF genes for improving antitumor response (169). OH2 (oncolytic herpes simplex virus 2) is generated to express GM-CSF transgene with deleted ICP34.5 and ICP47 genes expressions for improved tumor selective replication (244). A recombinant reovirus rS1-hsGMCSF was also generated for expressing the GM-CSF gene for stimulating antitumor activity (175). Vaccinia virus is also genetically altered to generate vvdd-tdTomato-hGMCSF by inserting GM-CSF and tdTomato fluorophore transgenes for dealing with immune suppression in pancreatic cancer (201). Deleted genes are written in red color with the (–) sign and added genes are written in green color with the (+) sign.

**Figure 4:**
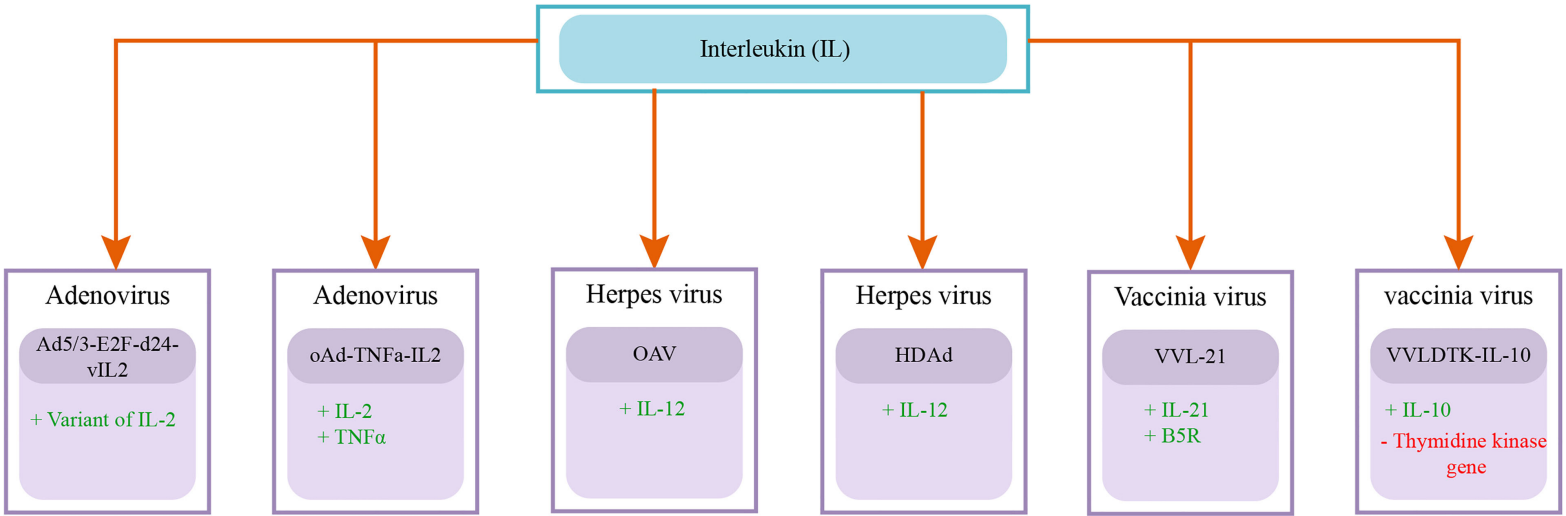
Recombinant Oncolytic Viruses armed with Interleukin (IL) genes: Interleukins function as a mode of communication between immune and non-immune cells. They play a crucial role in both innate and adaptive immune responses. These proteins also regulate cancer development by concurrently promoting cancer cell progression and evoking tumor targeted immune activity. Based on these properties, interleukins could be utilized in immunotherapy for impeding adverse effects and improved efficacy (245). Ad5/3- E2F-d24-vIL2 is a genetically altered adenovirus that expresses a variant of IL-2 for prolonged survival and eliciting immune response (135). Another recombinant adenovirus, oAd-TNFa-IL2, is specifically designed to express TNF-α and IL-2. The treatment of oAd-TNFa-IL2 combined with meso-CAR T cells resulted in curbed metastasis and improved immune activity in mouse models (139). Oncolytic viruses are genetically modified to express an antitumor cytokine, Interleukin 12. IL-12 expression aids in restricting tumor angiogenesis, in the differentiation of T-helper cells, which eventually promotes T cells directed cancer cell destruction (246). Oncolytic herpes simplex viruses are genetically modified by inserting IL-12 exogenous gene to generate OAV and HDAd. The administration of OAV caused high toxicity associated with overexpression of IL-12 in cancer cells, and treatment with HDAd resulted in comparatively slow release of IL-12 from the liver, which eventually caused restricted tumor growth (150). VVL-21 is a recombinant vaccinia virus, particularly designed to treat pancreatic cancer by incorporating B5R and IL-21 genes. The administration of VVL-21 combined with (α-PD1) triggered immune activity opposed to cancer cells (191). VVLDTK-IL-10, another recombinant vaccinia virus, is designed by omitting TK gene and inserting IL-10 exogenous gene for promoting antitumor response and prolonged survival (200). Interleukin (IL) gene along with other added genes written in green with + sign and deleted genes written in red with - sign.

Exogenous genes induction in oncolytic viruses improves the efficacy, specificity, and safety of the therapy. It is evident that oncolytic viruses are tumor-selective, as they rapidly and efficiently replicate in tumors (eventually causing cell death) compared to healthy tissue. Oncolytic viruses are genetically engineered to insert various favorable transgenes intending to improve therapeutic potential. The transgenes are effectively expressed in tumor sites only due to the tumor-selective nature of oncolytic viruses, which results in managing concerns related to systemic delivery (240). Interferons (IFN) are responsible to evoke antiviral and immunomodulatory properties. IFN*α* exhibited antiviral and antitumor effectiveness, both of these properties are independent of each other. It has been approved by the US FDA for treating certain malignancies either as a single agent or in combination with other drugs for improved efficacy (241). (242) genetically modified oncolytic vesicular stomatitis virus to express interferon-*β* (VSV-IFN-*β* ) for dealing malignant mesothelioma (pleura and peritoneum tumors). The expression of IFN-*β* transgene caused therapeutic improvements in murine mesotheliomas by promoting immune based effector mechanisms. Further, the safety profile also improved as INF-*β* expression also enhanced tumor specific viral replication, thus protecting from detrimental neurotoxicity. Consequently, the study suggested further investigation to improve VSV-IFN-*β* for clinical trials. Similarly, the expression of IFN*α* in genetically modified adenovirus OAd-hamIFN resulted in conducive clinical outcomes by showing considerably lessened toxicity along with improved antitumor activity (143). SJ-815 is a recombinant oncolytic vaccinia virus, specifically designed to express IFN*β* 1 and CES2 transgenes with deleted thymidine kinase-encoding (TK) gene. The combination of SJ-815 with irinotecan significantly restricts tumor growth in pancreatic cancer and resulted in increased survival in melanoma bearing mouse models (192). GM-CSF and interleukins are also important genes incorporated in oncolytic viruses as exogenous genes. Multiple oncolytic viruses armed with these genes are shown in **Figures 3**, **4**.

As stated earlier that oncolytic virus strains are tumor-colonizing, because of their capability of conditional replication in tumors. Nevertheless, the tumor stroma remains a challenge in oncolytic virus therapy. To overcome this and to promote antitumor response, the transgene expression approach is implemented. Intending to increase the efficacy and selectivity of adenoviruses (247), inserted palindromic E2F-binding sites into the endogenous E1A promoter in mice, by making sure to sustain virus functionality with slight growth in genome size. This study reported that insertion regulating E1A-Δ 24 resulted in an improved safety profile and cytotoxicity. Unsatisfactory spread and low potency of oncolytic adenovirus in tumor mass is the major limitation of cancer therapy. (248) tackled these issues and genetically engineered adenovirus (ICOVIR16) that expresses the envelope glycoprotein of gibbon-ape leukemia virus (GALV). The GALV expression resulted in improved cell fusion and the spread of the virus throughout tumor mass. The examination of ICOVIR16 for oncolytic properties in both *in-vivo* and *in-vitro* revealed boosted therapeutic efficacy. Mice having melanoma or pancreatic tumors were injected ICOVIR16 intravenously/intratumorally. It resulted in remarkably reduced tumor burden or complete elimination in some cases depicting enhanced oncolytic properties.

Numerous preclinical and clinical analyses of oncolytic viruses therapy along with Gemcitabine showed favorable outcomes (effective reduction of tumor size) in pancreatic cancer (149, 168, 215). Few preclinical studies are listed in **Table 3**, oncolytic viruses increase the effectiveness of Gemcitabine administration. Dose of Gemcitabine reduces in combination with oncolytic viruses, which can help in reducing the high dose toxicity of Gemcitabine. Evaluating the results of all the clinical trials of oncolytic viruses against pancreatic cancer. The combination therapy of adenovirus Ad5-DS (Ad5-yCD/mutTKSR39rep-ADP) along with chemotherapies (5-Fluorocytosine + Valacyclovir + Gemcitabine) display the highest median progression free survival (11.4 months) of LAPC patients. Detail of overall survival is not provided/reported by (134). The highest median overall survival rate so far reported in LAPC patients is 15.5 months in the clinical trial of HF10 in combination with Erlotinib and Gemcitabine (152). Both the studies show significant results and can be taken to the phase II trial for further evaluation of their effectiveness in LAPC. Overall the tumor cell specificity, and effectiveness of oncolytic viruses determined by previous studies compel to explore multiple options stated in this review. Likewise, utilizing viruses targeting disrupted pathways in pancreatic cancer cells can help in designing an efficacious therapy option against this fatal malignancy.

**Table 3:** Preclinical studies related to combination therapy of Gemcitabine with recombinant or native oncolytic viruses.

Sr. No	Viurses	Alterations in viruses	Other therapies	Study outcomes	reference
1	YDC002 Adenovirus	+ relaxin	–	Low (0.01-0.05µ M) dose shows effective results	jung2017oncolytic
2	Ad5-3Δ -A20T Adenovirus	–	–	high selectivity to cancer cells	(144)
3	shRNA-encoding adenovirus	–	TRAIL	tumor size regression	(149)
4	Myb34.5 herpes simplex virus	–		Low dose shows effective tumor size reduction	gayral2015targeted
5	oVV-Smac vaccinia virus	+ Smac gene	–	Increases apoptosis and cytotoxic effect of Gemcitabine	chen2019gemcitabine
6	VV-ING4 vaccinia virus	+ inhibitor of ING4 gene	–	Helps Gemcitabine to work more effectively as anticancer therapy	wu2017ing4
7	GLV-168 vaccinia virus	*β* -galactosidase, *β* -glucuronidase, and Ruc-GFP	Nab-paclitaxel	–	(195)
8	H-1PV Parvovirus	–	–	significant reduction of tumor size	(206) (207)
9	MeV Measles virus	–	–	> 50% reduction of tumor size	(215)
10	VSV- Δ M51-GFP Vesicular Stomatitis Virus	–	–		(230)

## Author Contributions

MN collected the data related to preclinical and clinical studies. MN and SA wrote the manuscript. All authors took part in the critical review of the manuscript. All authors contributed to the article and approved the submitted version.

## Funding

The authors received no financial assistance for conducting this research work. National University of Sciences and Technology (NUST), will pay the publication fee for this article.

## Conflict of Interest

The authors declare that the research was conducted in the absence of any commercial or financial relationships that could be construed as a potential conflict of interest.

## Publisher’s Note

All claims expressed in this article are solely those of the authors and do not necessarily represent those of their affiliated organizations, or those of the publisher, the editors and the reviewers. Any product that may be evaluated in this article, or claim that may be made by its manufacturer, is not guaranteed or endorsed by the publisher.
